# Hypoxia regulates RNA splicing of HIF targets

**DOI:** 10.18632/oncoscience.75

**Published:** 2014-08-21

**Authors:** Cheng-Jun Hu

**Affiliations:** Department of Craniofacial Biology, University of Colorado School of Dental Medicine, Aurora, CO 80045, USA

Hypoxia or reduced oxygen level is frequently observed in solid tumors. Hypoxia plays a significant role in solid tumor progression and metastasis by stabilizing hypoxia inducible factor (HIF) to activate gene transcription. The majority of human genes are alternatively spliced, producing RNA isoforms that code for functionally distinct proteins. Thus, it is unclear if HIF promotes tumor progression by increasing a particular RNA isoform or all isoforms of each HIF target gene. To address this question, we analyzed hypoxia response at RNA isoform levels [1, 2].

We report that following HIF activation, the levels of HIF target gene pre-mRNAs are increased; importantly, a particular RNA isoform of HIF target gene is preferentially induced by hypoxia [1, 2]. For example, fully spliced adrenomedullin (ADM) transcripts (ADM FL), not intron1, 2, 3 containing ADM transcripts (ADM I1-3) are increased under hypoxia although both ADM FL and I1-3 isoforms are equally expressed in normoxic cells [2]. Also, pyruvate dehydrogenase kinase 1 full-length (PDK1 FL), not exon-4 skipping isoform (ΔE4) is preferentially induced under hypoxia [1]. Although the functional difference between PDK1 FL and ΔE4 protein is not clear, increased production of ADM FL is functionally significant since ADM FL, not ADM I1-3 transcripts code for functional protein. These data indicate that hypoxia regulates HIF target gene expression by regulating two pathways, one to increase the levels of pre-mRNAs of HIF target genes, another to control pre-mRNA splicing of HIF target genes.

We also attempt to address the molecular mechanism underlying splicing regulation of HIF target genes. We show that HIF activity not hypoxia *per se* is necessary and sufficient to alter RNA splicing of HIF target genes such as *ADM* and *PDK1* [1, 2], indicating that transcription factor HIF directly or indirectly controls HIF target gene splicing. Using endogenous and splicing reporter genes, we show that transcription activation of *ADM* by HIF and any other transcription factors would promote ADM FL RNA isoform [2]. In addition, the *ADM* gene activation strength determines the efficiency of ADM FL production. These data indicate that intron removal from ADM pre-mRNA is directly regulated by HIF-mediated transcription activation. In contrast, increased PDK1 FL is only observed under condition when endogenous HIF target genes are activated [1]. Additional data indicate that increased levels of PDK1 FL require HIF-mediated activation of splicing factor(s). These data suggest that exon-4 inclusion of PDK1 is indirectly regulated by HIF activity. These results also suggest that the molecular mechanisms controlling intron-removal and exon-inclusion of HIF target genes could be different. It will be important to determine what are the HIF-regulated splicing factor(s) and how does the splicing factor regulates RNA splicing of HIF target genes.

Our recent studies also identify novel hypoxia-regulated genes/pathways that cannot be identified using traditional microarrays since hypoxia changes RNA splicing, not pre-mRNA levels of these genes [1]. We find that hypoxia mainly inhibits exon inclusion and/ or intron removal of non-HIF target genes [1]. Our data from hypoxia response studies is consistent with data from other stress response studies, in which most genes that are not involved in heat shock or salt stress response exhibit reduced RNA splicing due to increased intron-retention and/or exon exclusion [3, 4]. Interestingly, alternative splicing of these non-HIF target genes could be an important component of the hypoxic stress response. For example, genes involved in ATP binding and protein kinase activity are not hypoxia induced, but exhibit alternative splicing. Increased exon skipping of these non-HIF target genes in hypoxic cells could act to reduce ATP usage to maintain cellular ATP levels.

As described above, we find that hypoxia primarily promotes exon inclusion and/or intron-removal of HIF target genes but inhibits exon inclusion and/or intron removal of non-HIF target genes [1]. This opposing effect on HIF and non-HIF target genes for RNA splicing is similarly observed for gene transcription and protein translation, in which transcription and protein translation of non-HIF target genes are generally reduced while HIF target genes are transcriptionally induced and protein translation of genes involved in hypoxia response such as *HIF1A*, *HIF2A*, and *VEGF* are maintained [5]. These findings indicate that cells regulate multiple levels in gene expression in order to globally repress protein synthesis of non-HIF target genes, but selectively up-regulate hypoxic stress response proteins/pathways.

In summary, these studies indicate that RNA splicing is not a “housekeeping' process, but an active participant in gene expression. We hope that these observations will lead to a paradigm shift in our appreciation of the role of the RNA splicing regulation in gene expression control.

## References

[1] Sena JA (2014). Mol Cancer Res.

[2] Sena JA (2014). Mol Cancer Res.

[3] Ding F (2014). BMC genomics.

[4] Shalgi R (2014). Cell reports.

[5] Young RM (2008). JBC.

